# Effect of vaporizing cannabis rich in cannabidiol on cannabinoid levels in blood and on driving ability – a randomized clinical trial

**DOI:** 10.1007/s00414-023-03076-0

**Published:** 2023-08-26

**Authors:** Laura Egloff, Priska Frei, Kathrin Gerlach, Katja Mercer-Chalmers-Bender, Eva Scheurer

**Affiliations:** Institute of Forensic Medicine, Department of Biomedical Engineering, University of Basel, Pestalozzistrasse 22, 4056 Basel, Switzerland

**Keywords:** Cannabidiol, Δ^9^- tetrahydrocannabinol, Cognition, Driving ability, Road safety

## Abstract

The aim of this prospective, placebo-controlled, double-blind, randomized, cross-over study was to determine cannabinoid levels in blood and driving-related ability after single (S1) and repetitive (S2) vaporization of cannabis rich in cannabidiol (CBD) containing < 1% Δ^9^-etrahydrocannabinol (THC). Healthy adult volunteers (N_single_ = 27, N_repetitive_ = 20) with experience in smoking vapor-inhaled two low-THC/CBD-rich cannabis products both with < 1% THC (product 1: 38 mg CBD, 1.8 mg THC; product 2: 39 mg CBD, 0.6 mg THC) and placebo. Main outcomes were THC- and CBD-levels in whole blood and overall assessment of driving-related ability by computerized tests. Among 74 participants included, 27 (mean age ± SD, 28.9 ± 12.5 years) completed S1, and 20 (25.2 ± 4.0) completed S2. Peak concentrations and duration of detectability depended on the THC-content of the product. After single consumption THC dropped below 1.5 µg/L after 1.5 h, but was detected in some participants up to 5 h. Pairwise comparison of driving-related ability revealed no significant differences between low-THC/CBD-rich products (P1, P2) and placebo. Detection of THC after consumption of low-THC/CBD-rich cannabis might have legal consequences for drivers. Regarding overall driving-related ability, no significant differences were observed between the interventional products. This trial was registered with the German Clinical Trials Register (DRKS00018836) on 25.10.2019 and with the Coordination Office for Human Research (kofam) which is operated by the Federal Office of Public Health (FOPH) (SNCTP000003294).

## Introduction

Cannabis (*Cannabis sativa*) is the most widely consumed regulated substance worldwide [1]. Δ^9^-tetrahydrocannabinol (THC) is its main psychoactive constituent [2], while cannabidiol (CBD), another plant ingredient, is not considered to be intoxicating. Although international treaties often require the whole plant to be controlled under national drug laws [3], several countries make exceptions for plants with less than 0.2% [4], 0.6% [5] or 1% THC [6]. These regulations were intended to enable cultivation of industrial hemp, i.e. cannabis varieties used for the production of fibers and an assortment of commercial items. However, they also led to the sale of products with low THC- and high CBD-content, which could circumvent control by drug laws [7]. As recently evaluated by McGregor et al. [8], products containing CBD were indeed available (but not always legally) in several countries such as the US, Canada, Germany, Switzerland, Ireland, UK, and Japan. In Switzerland, the THC-threshold of 1% enables selling, possession, and consumption of so-called CBD-cannabis, legally classified as tobacco replacement, containing high quantities of CBD and < 1% THC [9].

Fitness to drive, hereafter referred to as driving-related ability, is defined as the momentary, time-limited, and event-related ability to safely participate in road traffic. Impairment can arise due to tiredness, influence of alcohol, medication, narcotics, amongst other reasons [10]. The impairing effects of THC on driving performance are well documented [11–17]. To ensure road safety, international-wide various regulatory frameworks have been established to manage driving under the influence of cannabis [18]. Based on the assumption that any THC use is incompatible with driving, several countries pursue a zero tolerance regime, when THC is detectable in a driver. However, non-zero limits, evidence of psycho-physical impairment, and other regulations are also in place [18, 19]. Switzerland pursues a zero tolerance regulation, which is implemented via two levels. THC-values up to a threshold of 1.5 µg/L in whole blood can have administrative and/or legal consequences, particularly when THC effects can be directly related to traffic law offences, accidents or other traffic events, and significant symptoms of restricted psycho-physical capacity. THC concentrations above the limit of 1.5 µg/L are punished as an offence independent of THC-specific symptoms [20]. Furthermore, at the time the law was put in place analytical instrumentation was much less sensitive and the use of different analytical techniques could result in positive testing in one laboratory, whilst in another laboratory the result could be negative. To overcome this issue and the lack of standardization in assessing drug related impairment, a legal threshold was introduced. Due to the standardized measurement uncertainty of ± 30% in Switzerland, a higher limit is applied in practice, i.e. at a THC-level of ≥ 2.2 µg/L driving inability is legally considered as proven.

Few studies on THC levels after the consumption of low THC/CBD-rich cannabis products are available. Additionally, the manner of administration in most studies involving inhalative consumption has been smoking [21–25] which is associated with lower bioavailability and reproducibility than vaporizing [26, 27]. Thus, blood concentrations of cannabinoids after smoking are not fully comparable to those after vaporizing. Following the consumption of cannabis varieties containing 0.8% to 0.94% THC, concentrations of THC were detected in whole blood of up to 4.5 ng/mL [21] and 6.8 ng/mL [22] in single subjects. A further study found THC-levels in serum of up to 10.8 µg/L after repeated exposure to a cannabis variety with 0.16% THC [23]. A study comparing placebo to a cannabis variety containing < 1% THC reported mean differences in THC plasma levels of 1.57 µg/L at 0 min after vapor inhalation [28]. Most recently, Gelmi et al. found levels of up to 102 ng/mL THC in capillary blood shortly after smoking of 1 g of a cannabis variety containing 0.9% THC [24].

Concerning the effects of low THC/CBD-rich cannabis products on driving performance, two reports focusing on the oral administration of comparable amounts of THC and CBD in patients suffering from multiple sclerosis found no significant effect [29, 30], and two studies showed that CBD does not prevent THC-induced impairment [31, 32]. A recent study found that vapor consumption of a cannabis variety containing 9% CBD (i.e. 13.75 mg) and < 1% THC did not lead to any significant impairment compared to placebo in an on-road driving test [28]. However, the dose applied in that study was smaller than in previous studies [21–23] and might, therefore, not be representative of typical recreational doses [24, 25]. Overall, there are not enough data on THC levels associated with potentially impairing effects that allow for science-based guidelines regarding the participation in road traffic after consumption of low THC/CBD-rich cannabis products. Due to its widespread use, guidelines are needed to inform and protect consumers who might be unaware of potentially exceeding a legal THC-limit or impairing their fitness to drive.

This study aimed to investigate THC and CBD levels in blood and whether there is evidence of impaired driving ability after consumption of CBD-rich cannabis with a THC-content < 1%. In study arm 1 (S1) volunteers underwent single consumption of two different cannabis products in a randomized, double-blind, cross-over, placebo-controlled design. Study arm 2 (S2) investigated frequent consumption (twice daily for 10 days) of the two cannabis-products in a randomized, double-blind, parallel design. Interventional products were administered using the Volcano medic® vaporizer, whereby product 1 released 38 mg CBD and 1.8 mg THC, and product 2 39 mg CBD and 0.6 mg THC. As THC-induced impairment is known to occur within initial hours after inhalation [11, 13–17, 33] a period of 5 h post consumption was monitored after each consumption in S1, and the final consumption on study day 10 in S2, respectively, by regular blood withdrawals. Driving-related ability was examined by a standardized and validated neuropsychological test system at 1 h and 3 h post consumption. The test system provided an overall assessment, which considers that performance deficits might be compensated through strengths in other ability ranges.

## Methods

The study was approved on 29.05.2019 by the ethics committee northwest/central Switzerland (BASEC-ID 2019–00639) and conducted in accordance with the Declaration of Helsinki ethical standards between September 2019 and August 2020 at the Institute of Forensic Medicine, University of Basel. This trial was registered with the German Clinical Trials Register (DRKS00018836) on 25.10.2019 and with the Coordination Office for Human Research (kofam) which is operated by the Federal Office of Public Health (FOPH) (SNCTP000003294).

Healthy volunteers were recruited via advertisement, webpage and word of mouth. Inclusion criteria were: aged 18 to 65 years; possession of a valid driver’s license; experience of smoking (tobacco and/or cannabis). Exclusion criteria were: insufficient German knowledge; psychiatric or physical disease (including addictive disorders); regular medication; pregnancy, breastfeeding or planned pregnancy; consumption of cannabis-products more than once weekly; non-compliance regarding alcohol abstention, controlled substances, cannabis products (including CBD-cannabis other than study intervention) for two weeks prior to and until the end of study participation and regarding abstaining from driving on study days. All volunteers meeting the inclusion criteria and without any obvious exclusion criteria underwent an interview for assessment of psychiatric disorders using the *Structured Clinical Interview for DSM-IV* (SCID-I; German SKID-I) [34, 35] screening questionnaire. All interviews were conducted by an experienced psychologist. All participants provided written informed consent prior to participation and were financially reimbursed. Participants and the study team supervising vaporization, testing of driving-related ability and conducting blood withdrawals, as well as laboratory staff analysing blood samples were blinded to the randomization list.

### Study intervention

Interventional products were prepared according to a computer generated randomization list by unblinded staff not further involved in the trial. The products were administered using the Volcano medic^®^ vaporizer (Storz & Bickel, Tuttlingen, Germany) employing two balloon fillings at 210 °C. In deviation from the original study protocol, according to which a dosage depending on body weight was planned, all subjects received the same dose. This was necessary because preliminary experiments indicated that the filling level of the vaporizer influences the release of cannabinoids, which would have led to results being difficult to compare. The following CBD-cannabis products were administered. Product 1: 300 mg of the cannabis variety *Harley Quinn* (14.6% CBD, 0.64% THC; Pure Production, Zeiningen, Switzerland); Product 2: 300 mg of the cannabis variety *V1 Haze* (4.3% CBD, 0.20% THC; Pure Production); Product 3: 300 mg of placebo-cannabis (cannabinoids < 0.2%; Bedrocan, Veendam, Netherlands). To adjust the CBD-content of product 2 to that of product 1, a drop pad (manufactured for use with the vaporizer) was fortified with 35 mg pharmaceutical-grade CBD (THC Pharm, Frankfurt am Main, Germany) as a 10% solution in Ph. Eur. ethanol (Merck, Buchs, Switzerland). Drop pads were dried at room temperature overnight and placed alongside product 2 in the vaporizer filling chamber. The above-mentioned vaporizer settings led to a release of approximately 38 mg CBD and 1.8 mg THC for product 1, and approximately 39 mg CBD and 0.6 mg THC for product 2 (incl. drop pad), as estimated by analysis of plant material and drop pad before and after use. Analyses of plant material and drop pad were conducted by gas chromatography coupled to a flame ionization detector (GC-FID). The method has been validated according to the guidelines of the Swiss Society of Legal Medicine (SGRM) and has previously proven suitable in proficiency testing [36].

### Study design, procedures and experimental sessions

The prospective, double-blind, randomized CBDrive study was conducted from September 2019 to August 2020 at the Institute of Forensic Medicine, University of Basel. Study personnel and investigators conducting test days, analysing blood samples and test results, and participants were blinded to the randomization list.

The study included two study arms. The placebo-controlled, cross-over study arm 1 (S1) consisted of three experimental sessions scheduled at least seven days apart to prevent carry-over effects. Before each experimental session, participants were tested for drug abuse using the Multi 12AC Dip Test in urine (nal von minden Drug-Screen, Germany). Participants tested positive for any substance were excluded from the study. In female participants, a pregnancy test (Alere hCG Cassette 25 mlU/mL, Abbott, Chicago, USA) was conducted at the first visit. An intravenous line was set up to collect baseline and all subsequent blood samples using 4 mL BD Vacutainer (1.5 mg/mL NaF and 3.0 mg/mL Na_2_EDTA; Becton Dickinson, Allschwil, Switzerland). Thereafter, participants consumed the interventional product assigned according to the randomization list which guaranteed an equal distribution of the sequence of the product in the study group. Participants were instructed to inhale every 20 s until both balloons were emptied. Duration of vaporization ranged from 3 to 23 min. At the last exhalation, the first blood sample was collected (0 min) followed by blood sampling at 5, 10, 15, 20, 30, 40, and 50 min, and 1, 1.5, 2, 2.5, 3, 3.5, 4, 4.5, and 5 h. A computerised neurocognitive test battery was conducted at 1 h and 3 h post inhalation. Upon the third completed visit, participants received financial reimbursement. Study arm 2 (S2) consisted of a preparation visit, 10 study days and a study visit on day 10. During the preparation visit, participants were tested as for S1 and received instructions on vaporizer use, urine collection and storage, and study visit schedules. Participants received the interventional product and vaporizer, including additional equipment (balloon, mouthpiece, lip piece), with instructions to vaporize the interventional product once every morning and evening. On study days 2, 4, 6, and 8, the evening vaporization took place on-site, with blood samples collected prior to, and immediately after vaporization. On study day 10, the evening vaporization was replaced by an on-site study visit identical to the S1 experimental session. Upon completion of the 10 study days, including the final experimental session, participants received financial reimbursement.

### Blood analyses

Analytical reference CBD and THC substances, and their deuterated analogues (CBD-D3 and THC-D3), were purchased from Lipomed (Arlesheim, Switzerland). Deuterated internal standard (final concentration 3 ng/mL) was added to 0.25 mL whole blood. Sample preparation was performed by automated on-line solid-phase extraction and derivatization with *N*-methyl-*N*-(trimethylsilyl) trifluoracetamide (Sigma Aldrich, St. Louis, USA) using a Multi Purpose Sampler II (Gerstel, Mühlheim, Germany). Prepared samples were analysed by GC coupled to tandem mass spectrometry (GC–MS/MS) using a Trace GC Ultra, equipped with a 30 m long Optima 5 MS GC-capillary (Macherey Nagel, Oensingen, Switzerland), coupled to a TSQ Quantum XLS mass spectrometer (both by Thermo Fischer, Waltham, USA). For both CBD and THC, the limits of detection were 0.15 µg/L, and the limits of quantification were 0.5 µg/L. The method was fully validated according to the guidelines of the SGRM and the German Society of Toxicological and Forensic Chemistry (GTFCh) [37] with proven suitability in proficiency tests.

### Overall driving-related ability

Driving-related ability was assessed using the standardised and validated Vienna test system neuropsychological test set *DRIVESTA* (Fitness to Drive Standard) [38] covering the five dimensions obtaining an overview, logical reasoning, concentration, stress tolerance, and ability to react. These dimensions are based on the Groeger’s action theory model regarding driving [39] and were grouped in three categories, namely planning the journey [40], executing the journey [41], and dealing with unforeseen situations [42–44]. The participant sat in an upright position in front of a portable computer and was instructed on how to perform the test. Participants had not been previously trained with the test system. However, as they had three study visits in S1, they already knew the procedure in study visits 2 and 3. Performance in all tracked dimensions was automatically recorded by the Vienna test system. An overall assessment of the respondent’s driving-related ability was provided by the system using a five-point scale, which considers that performance deficits might be compensated through strengths in other ability ranges. If registered deficits could not be fully compensated by strengths in other dimensions but only to a certain extent the overall rating was referred to as “partly compensable”.

### Outcomes and statistical analyses

Prespecified primary outcomes were the THC-concentration in whole blood samples collected at different time points after vaporization of CBD-cannabis and overall driving-related ability, as assessed in a computerised test set. Outcomes derived from the primary outcome data included maximum concentration (*c*_*max*_) and time of maximum concentration (*t*_*max*_) of CBD and THC.

All statistical analyses were conducted using the R environment for statistical computing (*R version 4.0.3*) [45]. Due to its pilot study character, sample size was not determined *a priori* by means of a statistical power calculation. Gender-related outcome was compared between groups with Pearson’s chi-square test. Age and years of education were compared with Welch’s two sample *t*-tests. THC- and CBD-peak levels in whole blood after consumption of product 1 and 2 were compared with paired sample *t*-test for S1 and Welch’s two sample *t*-test for S2, respectively. Ordinal mixed effects models from the ordinal package [46] in R for ordered categorical measures were used for analysing driving ability, with interventional product and time point as fixed effects and participant as random effect with varying intercept. In this study, the model was used to investigate if the driving ability was influenced by the interventional product (products 1 or 2 or placebo) or by the time period between consumption and the test. The parameter β refers to the estimate derived from the model, in this case to the ordinal rating categories of the overall driving ability provided by the Vienna test system. Positive β values indicate a positive correlation between impaired driving ability and the corresponding parameter, negative values indicate a negative correlation. The level of significance was set at *p* < 0.05. The 95% confidence intervals for the β estimates are given for an assessment of biomedical significance of the results.

## Results

**Fig. 1:**
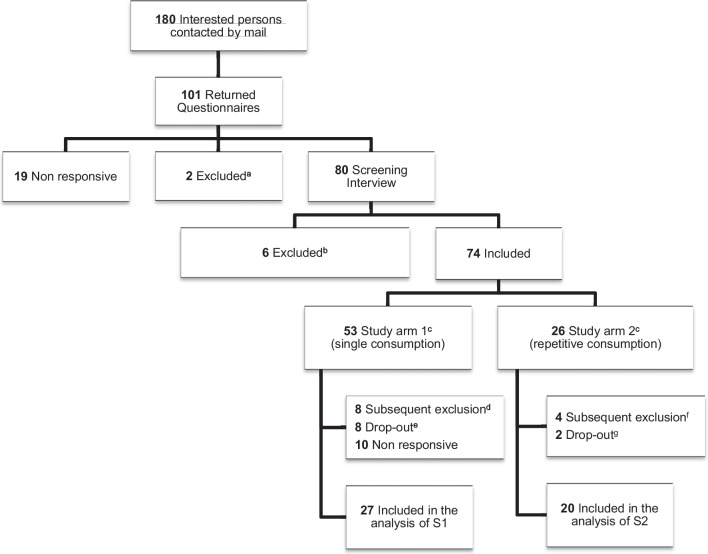
Flow of participants through the CBDrive study. ^a^ excluded after returning their questionnaires due to thyroid medication (*n* = 1) and diagnosed psychiatric disorder (*n* = 1). ^b^ excluded based on the screening interview due to insufficient knowledge of German (*n* = 1) or due to subsequent non-responsiveness regarding study enquiries (*n* = 5). ^c^ 5 persons volunteered for both study arms. ^d^ due to adverse event (subjective intolerance of study intervention; *n* = 1), impossible set up of intravenous line (*n* = 1), no experience with smoking (*n* = 1), diagnosed psychiatric disorder (*n* = 1), insufficient hearing ability (*n* = 1), no show and non-responsiveness (*n* = 1), excessive cannabis consumption (*n* = 1), and non-reliability (*n* = 1). ^e^ due to issues meeting the time requirements (*n* = 6) or personal reasons (*n* = 2). ^f^ due to adverse event (subjective intolerance of study intervention; n = 1), positive drug checking (*n* = 1), no show and non-responsiveness (*n* = 1), non-adherence to study protocol (*n* = 1). ^g^ due to work-related obligations with a necessity of driving (*n* = 2)

From 53 participants being enrolled in S1, and 26 in S2, 27 and 20 participants, respectively, were included in the analysis. Figure 1 shows the flow chart including the reasons for exclusions and drop-outs of enrolled participants.

Table 1 presents participants’ characteristics.

**Table 1:** Sociodemographic sample characteristics

	Study arm 1 (*n* = 27)	Study arm 2 (*n* = 20)
Age, years		
Mean ± SD	28.89 ± 12.47	25.15 ± 4.02
Median	23	24
Range	20–63	20–28
Sex, *n*		
Women	11	10
Men	16	10
Years of education	13.41 ± 1.97	13.85 ± 2.23

Numbers are presented as absolute numbers

No significant group differences were found between the S1 and S2 group regarding sex, age, and years of education (all *p* > 0.16). Women and men did not differ significantly regarding age (*p* = 0.12) or years of education (*p* = 0.94).

### Blood analyses

A total of 1268 blood samples, corresponding to 95% of planned blood samples, were analysed. Some samples were missing due to unfeasibility of blood withdrawals, particularly after repeated use of the venous catheter. After inhalation of placebo, no THC or CBD was detected in the blood samples. Table 2 summarizes the results regarding THC, i.e. *c*_*max*_, the detection rates, i.e. the percentage of samples above a respective concentration at a given time point, and last time points of THC detection. In all participants, *c*_*max*_ were found immediately after completed vaporization. Product 1 yielded significantly higher THC peak concentrations than product 2 (S1: product 1, *c*_*max*_ mean ± standard deviation, 11.8 ± 6.0 µg/L; product 2, 2.8 ± 1.5 µg/L; *t*(26) = 9.71, *p* < 0.001; S2: product 1, 14.5 ± 7.8 µg/L; product 2: 2.8 ± 1.4 µg/L; *t*(9.6) = 4.69, *p* < 0.001). After single product 1 consumption, THC-concentrations in whole blood dropped below 2.2 µg/L after between 30 and 40 min, and below 1.5 µg/L after 1.5 h, while THC was detected up to 5 h in 21% of participants. After 10 days of repetitive product 1 consumption, THC levels dropped below 2.2 µg/L after between 20 and 30 min, and below 1.5 µg/L after between 40 and 50 min. In 44% of the participants, THC was detected up to 5 h post consumption, i.e. in twice as many participants as compared to S1. After product 2 consumption, THC-concentrations ≥ 2.2 µg/L were reached immediately after vaporization, i.e. at 0 min, in both, S1 and in S2, and dropped below 2.2 µg/L within 5 min. THC was last detected in 12% and 10% of the participants at 1.5 h and 2.5 h after single and repetitive consumption, respectively.

**Table 2:** THC results of whole blood samples

Study arm	Product^a^	*c*_*max*_ [µg/L]	Detection rate of THC at 1 h, 3 h, 5 h			Last time of THC detection		
			≥ 2.2 µg/L	≥ 1.5 µg/L	≥ LOD	≥ 2.2 µg/L	≥ 1.5 µg/L	≥ LOD
S1: single consumption	1	1.9—28	0%^b^	7% (1 h) 0% (3 h, 5 h)	96% (1 h) 58% (3 h) 21% (5 h)	30 min (7%)	1.5 h (4%)	5 h (21%)
	2	< 0.5—6.9	0%^b^	0%^b^	19% (1 h) 0% (3 h, 5 h)	0 min (56%)	5 min (22%)	1.5 h (12%)
S2: repetitive consumption (day 10)	1	6.2—34	0%^b^	0%^b^	100% (1 h) 56% (3 h) 44% (5 h)	20 min (10%)	40 min (10%)	5 h (44%)
	2	1.2—5.8	0%^b^	0%^b^	30% (1 h) 0% (3 h, 5 h)	0 min (60%)	5 min (22%)	2.5 h (10%)

Abbreviations: *c*_*max*_ Maximum concentration; *THC* Δ^9^-tetrahydrocannabinol; *LOD* Limit of detection (0.15 µg/L)

^a^ product 1: 14.6% CBD, 0.64% THC (release: ca. 38 mg CBD, 1.8 mg THC); product 2: 4.3% CBD plus CBD doped drop pad (35 mg), 0.20% THC (release: ca. 39 mg CBD, 0.6 mg THC)

^b^ at 1 h, 3 h and 5 h

Table 3 provides an overview of the observed CBD-concentrations in whole blood. CBD was detected during the entire 5 h observation time in all participants of S1 and S2 after both, product 1 and 2 consumption, and in all blood samples collected prior to the last intervention of S2. *C*_*max*_ was reached immediately after vaporization. Despite the administered CBD amount in product 1 and 2 (ca. 38 mg and 39 mg, respectively) being almost identical, mean *c*_*max*_ in S1 after consumption of product 1 (173.1 ± 106.9 µg/L) was significantly higher compared to product 2 (120.4 ± 79.4 µg/L; *t*(26) = 3.89, *p* < 0.001). However, on day 10 of S2, product 1 (223.8 ± 121.2 µg/L) did not result in significantly higher *c*_*max*_ compared to product 2 (139.4 ± 99.6 µg/L; *t*(17.35) = 1.70, *p* = 0.107).

**Table 3:** CBD concentration in whole blood samples

Study arm	Product^a^	*c*_*max*_ [µg/L]	*c*_*5h*_ [µg/L]
S1: single consumption	1	39—520	< 0.5 – 4.9
	2	9.4—330	< 0.5 – 2.3
S2: repetitive consumption (day 10)	1	81 – 480	1.4 – 8.5
	2	40 – 350	1.2 – 3.6

Abbreviations: *CBD* Cannabidiol; *c*_*max*_ Maximum concentration; c_5h_ Concentration 5 h after vaporization

^a^ Product 1: 14.6% CBD, 0.64% THC (release: ca. 38 mg CBD, 1.8 mg THC); product 2: 4.3% CBD plus CBD doped drop pad (35 mg), 0.20% THC (release: ca. 39 mg CBD, 0.6 mg THC)

### Overall driving ability

Results from the overall driving ability assessment of the DRIVESTA test showed that a few participants showed driving ability impairments after consumption of product 1, product 2, and placebo, which were mostly either fully compensable or partly compensable, independent of the consumption pattern (single or repetitive) (Table 4). Although a small impairing effect could be detected for both products compared to placebo, which can be seen by the positive β estimate derived from the ordinal rating of the overall driving ability, neither of the interventional products had a significant influence on overall driving ability (Table 5). This can mainly be seen by the broad 95% confidence intervals for the β estimates ranging from values of about -1.6 to about 3.

**Table 4:** Results from the DRIVESTA test set

	Product 1^a^		Product 2^b^		Placebo	
	1 h	3 h	1 h	3 h	1 h	3 h
Study arm 1	*n* = 27	*n* = 27	*n* = 27	*n* = 27	*n* = 27	*n* = 27
Sufficient driving ability	22 (81.5%)	27 (100%)	25 (92.6%)	25 (92.6%)	24 (88.9%)	25 (92.6%)
Slightly impaired driving ability—performance deficiency can be fully compensated	2 (7.4%)	0	1 (3.7%)	0	1 (3.7%)	0
Impaired driving ability—performance deficiency can partly be compensated	2 (7.4%)	0	1 (3.7%)	1 (3.7%)	1 (3.7%)	1 (3.7%)
Impaired driving ability – performance deficiency cannot be compensated	0	0	0	1 (3.7%)	0	0
Missing	1 (3.7%)	0	0	0	1 (3.7%)	1 (3.7%)
Study arm 2	*n* = 10	*n* = 10	*n* = 10	*n* = 10	n.a.	
Sufficient driving ability	8 (80%)	9 (90%)	9 (90%)	10 (100%)		
Slightly impaired driving ability—performance deficiency can be compensated	2 (20%)	1 (10%)	1 (10%)	0		

Abbreviations: DRIVESTA, Fitness to Drive Standard; n.a., not applicable as no placebo condition was applied to study arm 2

^a^ Product 1 = 14.6% CBD, 0.64% THC

^b^ Product 2 = 4.3% CBD plus CBD doped drop pad, 0.20% THC

**Table 5:** Results from the ordinal mixed effects models

	β	S.E	z-value	*p*-value	*p*-value adjusted^a^	95% C.I	
						Lower bound	Upper bound
Product 1 – Product 2	0.319	1.05	0.305	0.761	0.950	-1.731	2.368
Product 1 – Placebo	0.854	1.11	0.767	0.443	0.724	-1.329	3.037
Product 2 – Placebo	0.535	1.11	0.484	0.628	0.879	-1.632	2.702
Time	-1.877	1.00	-1.875	0.061		-3.838	0.085

Abbreviations: *SE* Standard error; 95% *CI* 95% confidence interval

^a^ The Tukey method for comparing a family of 3 estimates was used for calculating adjusted *p*-values

## Discussion

Since the Swiss legal driving limit for THC is defined in whole blood, whole blood samples were analysed in this study [20]. Taking into account the blood/plasma-ratios of THC [47, 48] and CBD [49], higher concentrations are expected in plasma. Corresponding to previous studies [50], *c*_*max*_ of THC was found immediately after inhalation and was dose-dependent. THC-concentrations dropped below the Swiss legal limit of 2.2 µg/L (limit of 1.5 μg/L plus the harmonized measurement uncertainty of 30%) within 40 min, and below 1.5 µg/L within 2 h in all participants. The duration during which the limit was exceeded was also dose-dependent. Consumption patterns influenced the time window of detectability of THC but not the peak concentrations. Repetitive consumption of product 1 led to a higher detection rate at 5 h, while for product 2, detectability was prolonged, suggesting an apparent longer elimination time due to cumulative effects of THC after repetitive consumption [51, 52]. Additionally, it was observed that after single consumption of product 1, a THC blood concentration of ≥ 1.5 μg/L was detected after 1 h in a very small proportion of participants while this was not the case after repetitive consumption. However, this finding is not significant and might be coincidental based on the limited number of participants and individual variability. CBD was detected in all blood samples after both products, indicating a prolonged elimination period as previously described [53]. Despite products 1 and 2 containing similar amounts of CBD, product 1 led to significantly higher peak levels in S1. An explanation for a likely lower CBD uptake of product 2 might be an increased decomposition of CBD on the drop pad during heat exposure in the vaporizer as compared to plant material only [25].

To date, two reports have been published on THC- and CBD-levels in whole blood of a single participant after smoking of CBD-cannabis mixed with tobacco, but with comparable CBD- and THC-contents to product 1 [21, 22]. After smoking twice per day for ten days, maximum CBD- and THC-levels of 82.6 µg/L and 4.5 µg/L, respectively, were observed 15 min post-exposure [21]. Maximum levels of 105 µg/L CBD and 6.9 µg/L THC were found 18 min after the subject had smoked 4 cigarettes within 30 min [22]. Taking into account inter-individual variability and differences in interventional products and consumption patterns, our data confirm those previous findings.

In both study arms, assessment of overall driving-related ability revealed impairments in a few participants, which were either fully or partly compensable, respectively, and occurred regardless of the administered product (including placebo). The rating referred to as “partly compensable” implies that some deficits were observed which could not be fully compensated by strengths in other ability ranges, in contrast to the rating “fully compensable” where all deficits were fully compensated by strengths. No significant differences regarding driving ability were found between the CBD-cannabis products and placebo. Accordingly, three recent studies tested the influence of CBD-rich cannabis (all with < 1% THC and with 13.75 mg, 83 mg, and 148.92 mg CBD, respectively) and found no significant impairment of driving-related ability after consumption of the CBD-rich product compared to placebo or an uninfluenced basic testing, respectively [24, 25, 28]. Driving-related ability in these studies had been tested in an on-road driving test [28], which is rated to more closely resemble real driving situations, or by using neuropsychological test batteries for various cognitive and psychomotor dimensions similar to this study [24, 25] as well as vital signs [24].

THC is known to affect driving ability, at least for a certain period after consumption, whereby the impairment extent depends on dose as well as on drivers’ motivation to compensate deficits [28, 54–56]. In a meta-study, the frequency of performance impairments reportedly ceased within 3 h post consumption, while for high doses impairments persisting up to 5 h were reported [54]. However, driving can only be partly simulated in experimental laboratory studies [54] which might explain diverging results concerning driving ability after consumption of THC-rich cannabis [17, 28, 32].

### Limitations

Most of the participants were of younger age. However, according to data on illegal cannabis consumption from the Swiss Federal Office of Public Health [57], the 25–34 age group constitutes the largest proportion of consumers which is also in agreement with European Union figures [58]. Regarding gender, both Switzerland and the European Union report cannabis consumption prevalence as twice as high in men compared to women. Our study collective is, thus, representative in terms of age and gender distribution of the predominant cannabis consumer group. However, the study collective—designed as a pilot study—was small and therefore not representative of the general population.

As impairments of driving ability could also derive from general cognitive impairment of multiple domains, non-understanding of the testing instructions or performance inhibiting self-expectations, further neurocognitive tests should be conducted to ascertain any potential influence arising from the interventional products. Additionally, due to the tight blood sampling schedule during the first hour, the first neurocognitive examination using the test battery was performed at 1 h after consumption, a time interval which is also often seen in real cases of traffic controls. Thus, early signs of impairment may have been missed. However, the stimulating nature of some neurocognitive tests can also lead to suppression of impairments in contrast to monotone driving conditions, when activating stimuli are missing – particularly if the driver lacks the intrinsic motivation to compensate.

As THC was detected in the last blood samples collected 5 h after consumption in several cases, no final statement can be made on the duration of THC detectability. The spectrum of commercially available CBD-cannabis varieties differ not only in their CBD and THC content [9], but also in their overall profile of cannabinoids and phytochemicals [2]. These might have additional effects not investigated in this study. Similarly, other routes of administration, e.g. oral intake or smoking, consumption frequency, and dosing may result in a different outcome. The manner in which the cannabis dose was administered in this study does possibly not reflect the average typical route of administration, i.e. smoking of a cannabis/tobacco mixture. However, vaporizing is becoming increasingly popular and is known to provide a higher bioavailability of the active ingredients [26, 27]. Furthermore, with a vaporizer, cannabis can be consumed without tobacco and without inhalation of combustion products usually produced while smoking, putting the consumer at a perceived lower health risk. The likely higher bioavailability of THC and CBD through vaporization is seen as an advantage in this study as a higher likelihood of impairment can be expected with higher uptake of active ingredients. As no clear difference was observed in the group consuming low THC/CBD-rich cannabis in comparison to the placebo group, it is even more likely that by smoking the same product, no other outcome than that reported in this study should be expected. Vapor-inhalation is also an accepted manner of medical cannabis administration as dosing is thought to be more reproducible.

## Conclusion

Vaporization of both products (0.64% and 0.20% THC, releasing 1.8 mg and 0.6 mg THC, respectively) led to detectable amounts of THC in whole blood, whereby dose as well as consumption pattern had an influence on the duration and rate of the detection. Especially after the repeated consumption of the higher dose, THC was detected in the last blood sample drawn at 5 h post consumption in some participants, suggesting even longer detectability. Regarding driving, many countries pursue a zero tolerance for THC or have legal limit concentrations in place [18], while this is a matter of ongoing political and scientific debate in many countries including Switzerland. However, from the point of view of consumer protection, also consumers of low THC products should be aware of potential legal consequences. Regarding the Swiss legal driving limit, THC in whole blood had dropped below 2.2 µg/L at 40 min, and below 1.5 µg/L at 2 h. Thus, consumers with usage similar to that in this study should abstain from driving for at least 2 h after consumption, to prevent exceeding the Swiss THC legal limit. While there is no evidence that the investigated low-THC/CBD-rich cannabis products in the specified dosage and consumption manner impair overall driving ability, a potential impairment cannot be completely excluded as the used test battery may not be sensitive enough in detecting low levels of impairment, and drivers should exercise caution.

## Data Availability

The datasets generated and analysed during the current study are not openly available due to reasons of sensitivity, and restrictions apply to the obtainability of these data. Data are located in controlled access data storage at University of Basel. Data are, however, available from the authors upon reasonable request and with permission from the Federal Office of Public Health (FOPH). Neither the FOPH nor Storz & Bickel were involved in the design and conduct of the study, nor in the collection, management, analysis, and interpretation of the data. The authors have no competing interests to declare that are relevant to the content of this article.
